# Use of Pneumococcal Disease Epidemiology to Set Policy and Prevent Disease during 20 Years of the Emerging Infections Program

**DOI:** 10.3201/eid2109.150395

**Published:** 2015-09

**Authors:** Matthew R. Moore, Cynthia G. Whitney

**Affiliations:** Centers for Disease Control and Prevention, Atlanta, Georgia, USA

**Keywords:** pneumococcal disease, Streptococcus pneumoniae, bacteria, streptococci, vaccination, surveillance, epidemiology, antimicrobial resistance, Emerging Infections Program, EIP, Active Bacterial Core surveillance

## Abstract

Two decades ago, the Emerging Infections Program of the US Centers for Disease Control and Prevention implemented what seemed like a simple yet novel idea: a population- and laboratory-based surveillance system designed to identify and characterize invasive bacterial infections, including those caused by *Streptococcus pneumoniae.* This system, known as Active Bacterial Core surveillance, has since served as a flexible platform for following trends in invasive pneumococcal disease and studying vaccination as the most effective method for prevention. We report the contributions of Active Bacterial Core surveillance to every pneumococcal vaccine policy decision in the United States during the past 20 years.

## Abstract 2

This program has been a flexible platform for following trends of this disease and evaluating vaccine effectiveness.

*Streptococcus pneumoniae*, or pneumococcus, is the most common bacterial vaccine-preventable cause of death in the United States; globally pneumococcus is responsible for 476,000 deaths annually among children <5 years of age (*1*). Most of these deaths occur in developing countries. However, early efforts by the Emerging Infections Programs (EIPs) to track pneumococcus in the United States grew out of concerns regarding increasing antimicrobial resistance in the early 1990s. At that time, the Centers for Disease Control and Prevention noted an increase in drug-resistant strains reported through its passive, sentinel, hospital-based surveillance system (*2*) and determined that more intensive tracking of pneumococcal disease was needed.

Surveillance for invasive pneumococcal disease (IPD) began in 1995 as part of the EIP/Active Bacterial Core surveillance (ABCs) programs in California, Connecticut, Georgia, Maryland, Minnesota, Oregon, and Tennessee. IPD, defined for this program as isolation of pneumococcus from a normally sterile site, was chosen as the syndrome to be tracked because pneumococci identified from blood or cerebrospinal fluid are indicative of disease, whereas pneumococci from the respiratory tract might not be indicative, and because clinical practices associated with severe disease were unlikely to vary dramatically in different geographic areas. Audits of clinical laboratories, which can be performed during in-person visits or by electronic queries, aimed to ensure that all cases of IPD in EIP sites were ascertained. Extensive reviews of medical records enable investigators to ascertain underlying conditions, as well as discharge status. The population under surveillance in 1996 was >19 million, but sites were added in 1997, 2000, and 2004. Population growth within each site has increased the total population under surveillance to 31 million in 2014. ABCs have reported estimates of disease burden every year since 1998 (http://www.cdc.gov/abcs). A more detailed presentation of methods used in ABCs is provided by Langley et al. elsewhere in this issue (*3*).

Since the inception of ABCs, numerous publications have drawn heavily on primary analysis of ABCs pneumococcal data, and many others have incorporated secondary analyses of data published in peer-reviewed literature. Some of the most influential outputs have focused on basic descriptive epidemiology. For example, EIP/ABCs data on antimicrobial resistance among pneumococci causing IPD helped shape treatment policy for pneumonia and meningitis (*4**,**5*). A seminal paper containing data collected during 1995–1998 highlighted the increased risk for disease among children <2 years of age and adults ≥65 years of age, as well as substantial racial disparity (greater risk for black persons vs. white persons) in every age group (*6*). In addition, the analysis showed that 59% of disease among adults 18–64 years of age occurred in persons who had an indication for receiving 23-valent pneumococcal polysaccharide vaccine (PPV23). However, vaccine coverage was and remains unacceptably low. An estimated 48,000 cases (76%) of IPD and 5,300 deaths (87%) occurred annually among persons who were eligible for pneumococcal vaccines at that time. This analysis, which was conducted as pneumococcal conjugate vaccines were undergoing clinical trials, helped to highlight the need to include the conjugate vaccine in the US pediatric vaccine schedule and to improve use of PPV23 among adults.

Having a solid surveillance infrastructure in place has provided major opportunities for EIPs to conduct special studies. One of the earliest with key policy implications was the Preventability Study, which was designed to evaluate the extent to which addition of proposed new Advisory Committee on Immunization Practices (ACIP) indications for PPV23 might increase the proportion of IPD preventable through better immunization coverage (*7*). In 2000, the age for universal influenza vaccination was reduced from 65 to 50 years of age (*8*). This reduction raised the question of whether PPV23, which is frequently given to adults along with influenza vaccine, should also be administered to adults >50 years of age. In the Preventability Study, EIP investigators interviewed 1,705 adults who had recovered from IPD to identify all providers from whom they had received care. The EIPs then determined which patients had already received PPV23 and, among those who had not, which patients had at >1 ACIP indication for PPV23. Ultimately, the existing recommendations were proven to capture most adults with IPD, and the extant data were not sufficient to support reducing the age of universal vaccination with PPV23 (*7*).

Early EIP data served a major baseline for assessing the benefits of introduction of 7-valent pneumococcal conjugate vaccine (PCV7, Prevnar; Pfizer, Pearl River, NY, USA) in 2000. PCV7 was licensed on the basis of a randomized controlled trial in The Northern California Kaiser Permanente health care system, which demonstrated 97% efficacy against PCV7-serotype IPD when administered on a schedule of dosing at 2, 4, 6, and 12–15 months of age (*9*). The ACIP recommended use of PCV7 on that schedule (*10*), and the American Academy of Pediatrics issued similar recommendations (*11*). In 2001, a shortage of PCV7 led ACIP to recommend suspension of the booster (fourth) dose for healthy children (*12*) and, in 2003, a second shortage led to suspension of the third and fourth doses for healthy children (*13*). Although these shortages were unfortunate, they provided the EIPs with an opportunity to evaluate reduced-dose schedules, something which would have been challenging in the context of a randomized controlled trial.

The EIPs conducted a case–control study of PCV7 effectiveness during 2001–2003 and ultimately enrolled 782 case-patients and 2,512 controls (*14*). Effectiveness of >1 doses of PCV7 against PCV7-type IPD was 96%, and estimates of serotype-specific effectiveness were strikingly similar to those from the Kaiser trial (Table). However, because of the shortages, EIPs were able to demonstrate that virtually any PCV7 schedule with >2 doses in the first 6 months of life was 95% effective in preventing PCV7-type IPD. A schedule of 2 doses in the first 6 months, followed by a booster dose, was 98% effective. This 2 + 1 schedule was subsequently adopted widely in many countries. EIP/ABCs surveillance documented a 94% reduction in disease among children <5 years of age in the United States by 2003, in spite of the widespread shortages (Figure 1) (*15*).

**Table:** Comparison of serotype-specific effectiveness of PCV7 (EIP/ABCs case–control study) (*14*) with that of NCKP trial (*9*) against invasive pneumococcal disease*

Serotype	Vaccine effectiveness/efficacy, % (95% CI)	
	CDC/ABCs	NCKP trial 2000
All PCV7 types	Healthy: 96 (93–98); underlying illness: 81 (57–92)	94 (80–98)
4	93 (65–99)	NA
6B	94 (77–98)	86 (−11 to 100)
9V	100 (88–100)	100 (−142 to 100)
14	94 (81–98)	100 (60–100)
18C	97 (85–99)	100 (49–100)
19F	87 (65–95)	85 (32–98)
23F	98 (80–100)	100 (15–100)

*PCV7, 7-valent pneumococcal conjugate vaccine; EIP, Emerging Infections Program; ABCs, Active Bacterial Core Surveillance; NCKP, Northern California Kaiser Permanente; CDC, Centers for Disease Control and Prevention; NA, not available.

**Figure 1:**
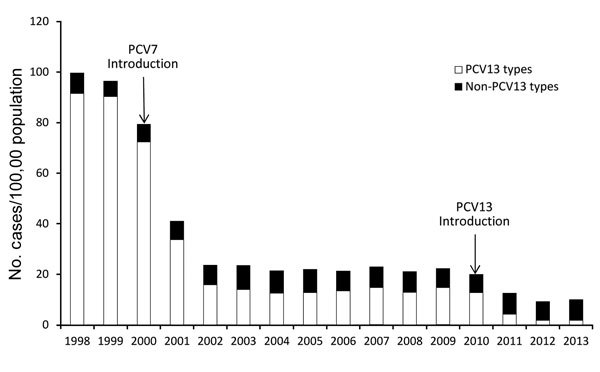
Incidence of invasive pneumococcal disease among children <5 years of age, caused by *Streptococcus pneumoniae* serotypes included in the 13-valent pneumococcal conjugate vaccine (PCV13) and by non-PCV13 serotype, Centers for Disease Control and Prevention Emerging Infections Program/Active Bacterial Core surveillance, 1998–2013.

Because the posterior nasopharynx had long been recognized as the reservoir for pneumococci, studies of asymptomatic colonization provided many insights into the dynamics of pneumococcal transmission. Multiple studies of the effects of pneumococcal conjugate vaccines on nasopharyngeal colonization demonstrated that vaccination prevents acquisition of vaccine-type pneumococci (*16*). This finding resulted in the hypothesis that widespread vaccination of children might reduce transmission to and ultimately, disease in adults. Herd protection conferred by PCV7 was far greater than predicted. Within the first 3 years of the PCV7 program in the United States, rates of PCV7-type IPD among adults began to decrease (*17*) and continued to decrease over subsequent years (Figure 2) (*18*). Whereas a primary driver of cost-effectiveness of PCV7 before introduction was the anticipated effect on otitis media visits among children, a key driver after introduction was the reduction in adult disease (*19*), something only identifiable through population-based surveillance. The cost per IPD episode averted without consideration of herd protection was $33,000, and the cost per episode averted with herd protection decreased to $5,500. This observation fundamentally changed the method for cost-effectiveness analyses of pneumococcal conjugate vaccines, not only in the United States (*20**,**21*) but also in other countries (*22**,**23*). A subsequent analysis, which incorporated the effect on pneumonia from non-EIP data sources, found PCV7 to be cost-saving (i.e., improved health outcomes at lower costs) (*20*).

**Figure 2:**
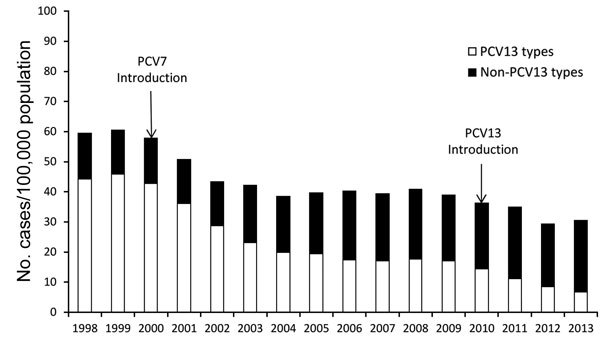
Incidence of invasive pneumococcal disease among adults >65 years of age caused by *Streptococcus pneumoniae* serotypes included in the 13-valent pneumococcal conjugate vaccine (PCV13) and by non-PCV13 serotype, Centers for Disease Control and Prevention Emerging Infections Program/Active Bacterial Core surveillance, 1998–2013.

During subsequent years, as shortages resolved, vaccine coverage increased and disease caused by PCV7 serotypes decreased, and EIPs detected an increase in rates of IPD caused by serotypes not included in PCV7. Even larger increases were described by other investigators in Alaska (*24*). A leading hypothesis was that these increases might represent serotype replacement, the process by which reductions in vaccine types open an ecologic niche for increases in nonvaccine serotypes in the nasopharynx, which ultimately lead to an increase in disease caused by nonvaccine serotypes. This phenomenon had been described in multiple randomized controlled trials of the effects of pneumococcal conjugate vaccines on nasopharyngeal colonization (*16*) but had never been described in the setting of invasive disease. Periodic increases and decreases in the incidence of invasive disease caused by certain serotypes, so-called secular trends, had been described for decades and this was the main hypothesis competing against serotype replacement in the early years after introduction of PCV7 (*25*).

Several antimicrobial drug–resistant strains were serotypes ultimately included in PCV7. Therefore, reductions in antimicrobial drug–resistant IPD were much anticipated and ultimately realized early on (*17*) and after several years of use of PCV7 (*26*). However, an observation compounding fears regarding serotype replacement was that serotype 19A was the non-PCV7 serotype with the greatest increase in incidence and that it was also associated with multidrug resistance (*27*). These findings suggested that inappropriate antimicrobial drug use was playing a role in the observed increases in non-PCV7 serotypes. Another hypothesis to explain the increase in serotype 19A after introduction of PCV7 was that genetic recombination events, whereby a PCV7 serotype could incorporate the genetic sequences of a non-PCV7 serotype, were occurring. So-called capsular switching might have contributed to increasing non-PCV7 serotype disease (*28*).

Ultimately, each of these mechanisms was shown to play a role. A systematic review of surveillance data from around the world, with EIP data being the primary contributor from North America, showed that increases in non-PCV7 serotypes were quite common in many settings and with many schedules of PCV7. However, in none of those settings did the increases in non-PCV7 IPD overshadow the reductions in PCV7-type IPD in children <5 years of age (*29*). Secular trends appeared to be a minor contributor in the United States, where epidemic serotypes 1 and 5 are relatively uncommon. Antimicrobial drug use probably influenced selection of antimicrobial drug–resistant strains among those serotypes (e.g., 19A) destined to cause replacement disease (*30**,**31*). Finally, capsular switching clearly occurred but played a minor role in the increases in non-PCV7 serotypes (*28*). In some settings, improvements in surveillance methods at or after the time of PCV7 introduction might have falsely enhanced the increase in IPD caused by nonvaccine serotypes (*32*).

Worries concerning serotype replacement were tempered to an extent by the anticipated licensure of PCV13 in 2010. PCV13 included the same serotypes as PCV7 plus 6 additional types: 1, 3, 5, 6A, 7F, and, most important, 19A, the dominant replacement serotype worldwide. The US Food and Drug Administration licensed PCV13, and ACIP voted to recommend its use for children in February 2010 (*33*). PCV13 had large and immediate effects, in part because it was licensed on the same schedule as PCV7, which enabled rapid swapping out of PCV7 for PCV13. Coverage increased rapidly, and by the end of 2011, EIPs identified reductions in PCV13-type IPD, not only among children but also among adults (*34*). In the short term, the benefits of PCV13 appeared comparable with those of PCV7 and have resulted in large reductions in serotypes that caused most replacement disease after widespread PCV7 use (19A and 7F). Nonetheless, more time is needed to determine whether remaining nonvaccine types will cause extensive replacement disease.

The PCV7 immunization program for children also benefited persons with immunocompromising conditions. After PCV7 introduction, rates of IPD caused by PCV7 serotypes among adults with HIV infection decreased substantially. When PCV13 was licensed for adults in 2011, ACIP discussed the possibility of recommending that vaccine for adults with immunocompromising conditions, including HIV (*35*). Rates of PCV7-type IPD among HIV-infected adults had remained extremely high despite having decreased from their pre-PCV7 baseline (*36*). Around the same time, a randomized controlled trial of PCV7 in HIV-infected adults in Malawi showed PCV7 to be 74% effective in preventing PCV7-type IPD. ACIP considered, among others, these 2 factors—high remaining burden of PCV7-type IPD among HIV-infected adults in the EIPs and demonstrated efficacy of PCV7—in ultimately recommending PCV13 for immunocompromised adults (*37*). On the basis of similar EIP data on disease burden among adolescent children, the ACIP ultimately recommended PCV13 for that population as well (*38*).

The most recent and perhaps widest-ranging change in ACIP recommendations came about in August 2014, when PCV13 was recommended for every adult >65 years of age in the United States (*39*). After initially refraining from recommending PCV13 for this group (*35*), the ACIP reviewed extensively results of a randomized controlled trial in the Netherlands, which became available in early 2014 and showed that PCV13 was 76% effective in preventing PCV13-type IPD among persons >65 years of age and 45% effective against non-invasive pneumonia caused by PCV13 serotypes (*40*). However, if there were no PCV13-type disease remaining, the ACIP might not have ever recommended the vaccine for this population of 44 million adults. Instead, data from the EIPs were instrumental in demonstrating that, despite major reductions in rates of PCV7- and PCV13-type IPD among adults, the remaining disease burden was sufficiently high that a universal, age-based recommendation was cost-effective in the short term (*39*).

Pneumococcal disease epidemiology has changed substantially in the United States in the past 20 years because of new prevention measures. Disease has decreased, first as a result of PCV7 introduction and, most recently, as a result of PCV13 introduction. EIPs have documented the effects of this vaccine on disease in children, disease in adults, and antimicrobial drug resistance and have provided data that helped to refine vaccine policy in the United States and elsewhere. The EIPs have elucidated the complex mechanisms at play when increases in nonvaccine-type disease are observed after reductions in vaccine-type disease and when antimicrobial drug resistance increases in response to inappropriate antimicrobial drug use and decreases in response to vaccination. In addition, the EIPs have contributed in fundamental ways to every pneumococcal vaccine recommendation in the United States since 2000. For these reasons, the EIPs have reason to celebrate their 20th anniversary.
